# The clinical application of metagenomic next-generation sequencing for invasive pulmonary aspergillosis in neutropenic patients: a multicenter retrospective study in the ICU

**DOI:** 10.3389/fcimb.2026.1878097

**Published:** 2026-08-03

**Authors:** Jing Tang, Jiahao Deng, Kai Guo, Yong Song, Junjie Zhao, Xiaojing Zhang, Youqin Yan, Lingmin Yuan, Yi Zhang, Canhu Qiu, Jian Luo, Honglong Fang, Jiancheng Zhuge

**Affiliations:** 1 Jin Hua Graduate Joint Training Base, Zhejiang Chinese Medical University, Hangzhou, Zhejiang, China; 2 Emergency and Critical Care Center, Department of Emergency Medicine, Zhejiang Provincial People’s Hospital (Affiliated People’s Hospital, Hangzhou Medical College), Hangzhou, Zhejiang, China; 3 WillingMed Technology Beijing Co., Ltd, Beijing, China; 4 Department of Critical Care Medicine, People’s Hospital of Changshan County, Quzhou, Zhejiang, China; 5 Department of Critical Care Medicine, Longyou County People’s Hospital, Quzhou, Zhejiang, China; 6 Department of Critical Care Medicine, Quzhou Kecheng People’s Hospital, Quzhou, Zhejiang, China; 7 Department of Critical Care Medicine, Jiangshan People’s Hospital, Quzhou, Zhejiang, China; 8 Department of Critical Care Medicine, The Quzhou Affiliated Hospital of Wenzhou Medical University, Quzhou People’s Hospital, Quzhou, Zhejiang, China; 9 Department of Emergency Medicine, Quzhou Hospital of Traditional Chinese Medicine, Quzhou, Zhejiang, China

**Keywords:** antifungal therapy, invasive pulmonary aspergillosis, metagenomic next-generation sequencing, neutropenia, treatment outcome

## Abstract

**Background:**

Early initiation of targeted antifungal therapy is critical for improving outcomes in neutropenic patients with invasive pulmonary aspergillosis (IPA) in the intensive care unit (ICU). Although metagenomic next-generation sequencing (mNGS) is valuable for pathogen detection, its clinical value in IPA patients with neutropenia remains unclear.

**Methods:**

This multicenter retrospective study included patients clinically diagnosed with invasive pulmonary aspergillosis (IPA). All patients underwent both conventional microbiological tests (CMTs) and metagenomic next-generation sequencing (mNGS) of bronchoalveolar lavage fluid (BALF). Based on neutrophil status, patients were stratified into neutropenic and non-neutropenic groups and further divided into mNGS-guided and CMT-guided groups according to the antifungal treatment strategy.

**Results:**

mNGS demonstrated higher pathogen detection rate than conventional microbiological tests (CMTs) in both neutropenic and non-neutropenic patients with invasive pulmonary aspergillosis (IPA). It also identified a broader pathogen spectrum and a higher proportion of mixed infections. Overall, IPA patients in the mNGS-guided group had lower 28-day mortality compared with the CMT-guided group (23.17% vs. 43.75%, P = 0.04). Multivariate analysis indicated that mNGS-guided therapy was associated with reduced 28-day mortality (adjusted OR = 0.329, 95% CI: 0.111–0.974, P = 0.045). A significant interaction between treatment strategy and neutrophil status was observed (adjusted P = 0.002). In subgroup analysis, the survival benefit of mNGS-guided therapy was mainly observed in neutropenic IPA patients, who achieved higher rates of appropriate antifungal therapy and lower mortality, whereas no significant intergroup difference was found among non-neutropenic IPA patients.

**Conclusion:**

mNGS-guided antifungal therapy significantly reduced 28-day mortality in neutropenic IPA patients, whereas no clear effect was observed in non-neutropenic patients. These findings highlight the potential clinical value of mNGS in guiding antifungal therapy in neutropenic IPA patients.

## 1 Introduction

With the widespread use of chemotherapy, organ transplantation, and immunosuppressive therapies, the number of immunocompromised patients, including those with neutropenia, continues to increase (Duo and Deshpande, 2025; Martin-Loeches et al., 2025).

Invasive pulmonary aspergillosis (IPA) is a severe, fast-progressing fungal infection with high mortality, especially among neutropenic patients (Kousha et al., 2011; Elkhapery et al., 2025; Kim et al., 2025). Once seen mainly in neutropenic patients, IPA now has rising rates in critically ill non-neutropenic patients, such as those with COPD, liver cirrhosis, or on corticosteroids (Liu et al., 2025; Liu et al., 2026). IPA patients may differ markedly in clinical presentation, disease progression, radiological findings, and treatment response according to neutrophil status (Liu et al., 2021). Therefore, stratification based on neutrophil status is necessary to optimize diagnostic and therapeutic strategies and improve patient outcomes (Wang et al., 2024; Gao et al., 2026). Early and accurate pathogen identification, followed by timely initiation or adjustment of targeted antifungal therapy, is critical for improving the clinical prognosis of IPA. However, in routine clinical practice, pathogen-based diagnosis remains challenging (Inoue et al., 2022; Feng et al., 2025). Conventional microbiological methods are limited by prolonged turnaround time and suboptimal sensitivity.

Metagenomic next-generation sequencing (mNGS) is a novel high-throughput technology that requires no predefined targets and can simultaneously detect bacteria, fungi, and viruses in clinical specimens (Miller and Chiu, 2021; Zhao et al., 2026). In contrast to traditional methods, mNGS offers higher sensitivity, broader pathogen coverage, and shorter turnaround times. These advantages may enhance IPA diagnosis and support early intervention. However, despite its promise, the clinical value of mNGS in ICU patients with invasive pulmonary aspergillosis remains under-evaluated, especially across varying neutrophil statuses. Therefore, this study was conducted in patients with varying neutrophil counts, with a particular focus on evaluating the clinical value of mNGS in IPA patients with neutropenia.

## 2 Materials and methods

### 2.1 Study design and participants

This study used a multicenter retrospective design. A total of 122 ICU patients with clinically diagnosed IPA were enrolled between January 2022 and January 2026 from Quzhou People’s Hospital, Quzhou Traditional Hospital, Chinese Medicine Hospital, Changshan County People’s Hospital, Jiangshan City People’s Hospital, Longyou County People’s Hospital, and Kecheng District People’s Hospital. As histopathological confirmation (e.g., lung biopsy) was not available, all cases were classified as clinically diagnosed IPA according to the 2020 European Organization for Research and Treatment of Cancer/Fungal Disease Study Group consensus (Bassetti et al., 2020): Host factors (meet any one criterion: recent neutropenia with ANC <500/μL persisting ≥10 days, hematologic malignancy, allogeneic hematopoietic stem cell transplantation, long-term glucocorticoid use equivalent to ≥0.3 mg/kg/d prednisone for ≥3 weeks, T-cell immunosuppressive agent use within 90 days, or hereditary immunodeficiency); Clinical Features (meeting any of the following on chest imaging: nodular or consolidated opacities with cavitation, halo sign or crescent sign, multiple patchy consolidations or tree-in-bud sign);. Microbiological evidence was defined according to the 2020 EORTC/MSGERC criteria. Any of the following findings was considered mycological evidence: (i) positive culture and/or histopathological examination for Aspergillus; (ii) positive galactomannan (GM) test, defined as at least one of the following: serum or plasma ≥ 1.0, bronchoalveolar lavage fluid (BALF) ≥ 1.0, serum or plasma ≥ 0.7, or BALF ≥ 0.8; (iii) at least two positive PCR results in plasma, serum, whole blood, or BAL fluid, or at least one positive PCR result in both blood (plasma/serum/whole blood) and BAL fluid; and (iv) Aspergillus spp. isolated by culture from sputum, BALF, bronchial brushings, or aspirates. In this study, BALF metagenomic next-generation sequencing (mNGS) was additionally incorporated as an adjunct microbiological method to improve pathogen detection. This modification did not alter the original host factor or radiological criteria of the EORTC/MSGERC framework. mNGS positivity was defined as ≥50 unique reads assigned to a single Aspergillus species, according to previously published clinical studies (Shi et al., 2023); results below this threshold were not considered sufficient microbiological evidence for the diagnosis of invasive pulmonary aspergillosis (IPA). The final diagnosis required integration of host factors, imaging findings, and clinical response, in accordance with the EORTC/MSGERC consensus criteria.

Neutropenia is defined as a neutrophil count <500/mm³ at the time of Aspergillus infection diagnosis or within the preceding month (Gutiérrez-Villanueva et al., 2025).

**Figure 1:**
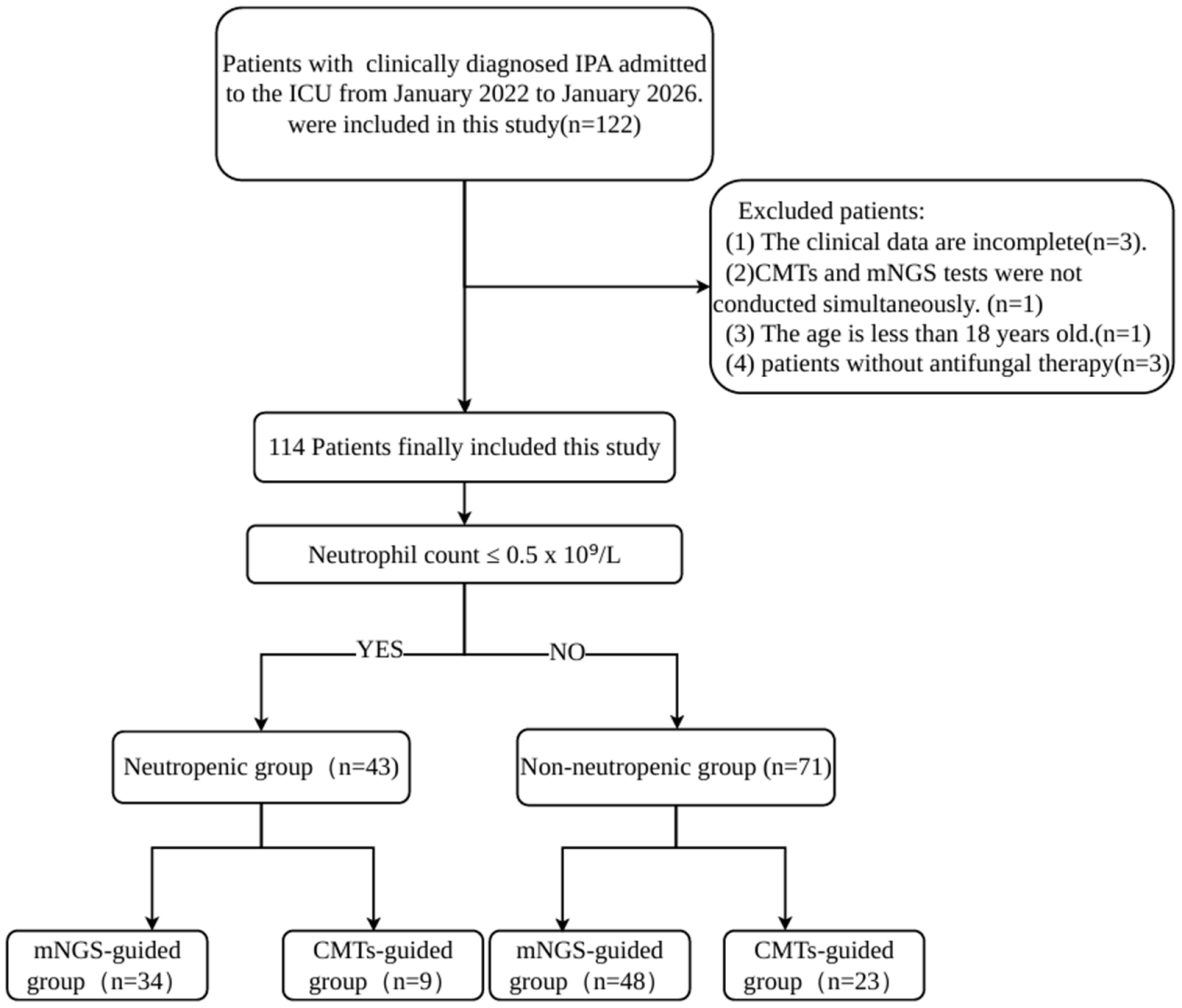
Study flow diagram.

Inclusion criteria include: meeting the clinically diagnosed IPA; undergoing bronchoalveolar lavage with simultaneous submission of lavage specimens for routine culture, Gram staining/Gram-negative bacilli (G/GM) test, and mNGS; and completion of systematic testing for other potential pathogens (including but not limited to bacterial culture, acid-fast staining, Polymerase Chain Reaction (PCR)for Pneumocystis jirovecii, Mycobacterium tuberculosis, and cytomegalovirus). Exclusion criteria: incomplete clinical data; non-concurrent submission of routine microbiological testing and mNGS; age <18 years; without antifungal treatment. As shown in the flowchart in **Figure 1**, after screening for inclusion and exclusion criteria, a total of 114 cases were included in the analysis (**Figure 1**).

### 2.2 Sample collection and conventional microbiological testing

After contraindications were excluded, bronchoscopy was performed by trained physicians following standardized aseptic protocols to obtain bronchoalveolar lavage fluid (BALF) samples BALF specimens were used for both metagenomic next-generation sequencing (mNGS) and conventional microbiological tests (CMTs). In addition, blood, sputum, and other relevant clinical specimens were collected simultaneously for CMTs, as appropriate. CMTs included bronchoalveolar lavage fluid (BALF) culture, sputum culture, blood culture, quantitative polymerase chain reaction (qPCR), T-cell spot test (T-SPOT), GeneXpert, the (Duo and Deshpande, 2025; Elkhapery et al., 2025)-β-D-glucan (G) test, and the galactomannan (GM) test. T-SPOT and GeneXpert were used to detect Mycobacterium tuberculosis.

### 2.3 Metagenomic next-generation sequencing workflow

DNA/RNA Extraction: Centrifuge BALF samples at 4 °C Centrifuge at 12075×g for 5 minutes. Collect 500 μL supernatant. Extract genomic DNA and RNA using the PathoXtract^®^ Universal Pathogen Extraction Kit (Willingmed, China), strictly following the manufacturer’s instructions.

### 2.4 Library construction and sequencing

cDNA was synthesized from RNA using the PathoXtract^®^ RNA Purification Kit (WYCM06601D-24, Willingmed, China). Equal amounts of DNA and RNA were then mixed thoroughly, and library construction was performed using the PathoLib™ Genome Fragmentation Kit (WYLM044032D-96, WillingMed, China) and the PathoLib™ Genome Library Enrichment Kit (WYLM04407S-96, WillingMed, China). Library quality was assessed using a Qubit Fluorometer and Agilent 2100 Bioanalyzer before sequencing on the MGISEQ-200 platform, with each sample yielding ≥10 million reads.

All BALF mNGS specimens collected from all participating multicenter sites were delivered to WillingMed for unified testing, adopting identical library kits, Illumina MGISEQ-200 sequencing platform and standardized bioinformatic pipelines across all samples. For conventional microbial tests (CMTs), all centers followed the same unified standard operating procedures for culture, smear and antigen detection to minimize inter-center technical differences and ensure multicenter data comparability.

### 2.5 Bioinformatics analysis

Raw data were filtered using Trimmomatic v 0.40 (Bolger et al., 2014). to remove low-quality, adapter-contaminated, low-complexity, and undersized sequences, yielding high-quality data. Sequences were aligned to the human reference genome using Bowtie 2 v 2.4.3 (Langmead and Salzberg, 2012)to remove human sequences. Remaining sequences were classified by aligning them to NCBI bacterial, fungal, viral, and parasitic databases using Kraken2 v2.1.0 (Wood et al., 2019).

### 2.6 mNGS positivity criteria

Quantification was performed using the pathogen-specific sequence count per 10 million sequences (RPTM). The RPTM ratio was defined as the sample RPTM divided by the negative control RPTM. For species or genera with a microbial classification value < 1 in the negative control, this ratio was set to 1. Viral positivity threshold: RPTM ratio ≥3 per million sequences. Bacterial and fungal positivity threshold: RPTM ratio ≥20; Special pathogens (e.g., Cryptococcus, Mycobacterium, NTM): RPTM ratio ≥1 is considered positive. To exclude interference from colonizing bacteria, detected strains were compared against the hospital background microbial database; strains matching common colonizing bacteria in the database were excluded from analysis.

Mixed infection was defined as the identification of two or more clinically relevant pathogens from the same respiratory specimen.

### 2.7 Consistency analysis of mNGS and CMTs results

To further compare the differences between the two methods in detecting Aspergillus and other pathogenic microorganisms, among patients with positive findings on both mNGS and CMTs, the following criteria were established, and the detection results were visualized in a composite pie chart format:

1. Complete consistency: Both mNGS and CMTs detected Aspergillus infection, and the detection results for other pathogenic microorganisms were also entirely consistent in both methods.
2. Partial consistency: Both mNGS and CMTs detected Aspergillus infection; however, differences were observed between the two methods in the detection of other microorganisms, with one method identifying additional pathogens not detected by the other.

Evaluation of the Impact of mNGS and Conventional Microbiological Testing on Anti-infective Treatment Adjustment and Clinical Outcomes.

### 2.8 Study grouping

Patients were categorized according to the diagnostic modality that primarily contributed to antifungal treatment decisions based on documented clinical records. All included patients had at least one positive microbiological result obtained by BALF mNGS or conventional microbiological tests (CMTs).

The mNGS-guided therapy group included patients in whom BALF mNGS findings primarily contributed to antifungal treatment initiation or modification, including initiation, discontinuation, escalation, de-escalation, or switching of antifungal agents, as documented in medical records.

The CMTs-guided therapy group included patients in whom antifungal management was mainly determined by CMTs results combined with routine clinical assessment, and mNGS findings did not result in further modification of antifungal therapy, including initiation, discontinuation, escalation, de-escalation, or switching of antifungal agents.

#### 2.8.1 Sensitivity analysis using objective temporal classification criteria

To further evaluate the robustness of the primary classification and minimize potential classification bias, a sensitivity analysis was conducted using pre-specified temporal criteria.

Patients were reclassified according to the chronological sequence between microbiological reporting and antifungal treatment modification.

The mNGS-guided therapy group was defined as patients who met both of the following criteria:

1. BALF mNGS results became available before antifungal treatment modification; and
2. medical records explicitly documented that antifungal treatment adjustment was based on mNGS results.

The CMTs-guided therapy group was defined as patients who met any of the following criteria:

1. antifungal management was determined based on available CMTs results before mNGS reporting;
2. antifungal treatment modification occurred before mNGS results became available; or
3. mNGS results became available but did not lead to further modification of antifungal therapy.

For patients with positive results from both mNGS and CMTs, group allocation was determined according to the temporal sequence of microbiological reporting and antifungal treatment modification rather than microbiological positivity alone.

### 2.9 Evaluation criteria and definitions

Appropriate antifungal therapy was defined as the timely initiation of an antifungal agent with activity against Aspergillus spp, administered at an appropriate dose and route, and subsequently adjusted based on microbiological findings, antifungal susceptibility testing, and patient-specific clinical factors, in accordance with current international guidelines (e.g., Infectious Diseases Society of America [IDSA] and European Society of Clinical Microbiology and Infectious Diseases [ESCMID] guidelines) (Patterson et al., 2016).

Turnaround time (TAT) was defined as the interval from receipt of the clinical sample in the laboratory to the final reporting of results (Dawande et al., 2022).

To ensure the reliability of group classification and diagnostic assessment, two clinicians with over 10 years of ICU experience (Fang Honglong and Zhuge Jiancheng) independently reviewed and comprehensively interpreted patient clinical characteristics, routine test results, mNGS findings, laboratory data, and imaging studies (e.g., chest CT). Notably, both adjudicators were fully blinded to all patient follow-up outcomes (including 28-day mortality) during the entire evaluation process to avoid assessment bias. Any discrepancies were resolved by consensus, and consistency between clinical diagnoses and test results was ultimately confirmed.

## 3 Data analysis methods

Statistical analysis was performed using SPSS, with graphs generated using OriginPro and R. Continuous variables were assessed for normality using the Kolmogorov–Smirnov test and presented as mean ± standard deviation or median (interquartile range), as appropriate. Group comparisons were performed using independent-samples t-tests or Mann–Whitney U tests for continuous variables, and chi-square or Fisher’s exact test for categorical variables. Kaplan–Meier survival analysis was used to estimate 28-day survival, and differences between groups were compared using the log-rank test. Multivariate logistic regression was used to identify independent predictors of 28-day mortality, with adjustment for neutrophil status and SOFA score. An interaction term between neutropenic status and mNGS-guided therapy was added to assess effect modification. All tests were two-sided, with P < 0.05 considered statistically significant.

## 4 Results

### 4.1 Baseline characteristics of ICU patients with invasive pulmonary aspergillosis

A total of 114 ICU patients with clinically diagnosed IPA were included, comprising 43 neutropenic and 71 non-neutropenic patients. As shown in **Table 1**, baseline characteristics of ICU patients with IPA are stratified by neutropenic status (**Table 1**).

**Table 1:** Baseline characteristics of ICU patients with IPA stratified by neutropenic status.

Characteristics	Neutropenic group (n=43)	Non-neutropenic group(n=71)	*P* value
Age years (M, IQR)	67 (58–77)	76(70-81)	0.001
Female (%)	10(23.26%)	10(14.08%)	0.309
Fever, °C (M, IQR)	37.2(36.80-38.00)	37.00(36.70-37.40)	0.048
Comorbidity			
Hypertension, (n, %)	28(65.12%)	39(54.93%)	0.329
Diabetes, (n, %)	11(25.58%)	22(30.99%)	0.671
Heart disease, (n, %)	7(16.28%)	21(29.58%)	0.123
COPD (n, %)	21(48.84%)	53(74.65%)	0.008
Kidney disease (n, %)	9(20.93%)	36(50.70%)	0.002
After chemotherapy (n, %)	18(41.86%)	3(4.23%)	0.000
Hematologic disease (n, %)	22(51.16%)	21(29.57%)	0.028
Immunocompromised (n, %)	31(72.09%)	48(67.61%)	0.679
Clinical laboratory data			
WBC, ×10^9^/L (M, IQR)	4.85(3.50-7.45)	12.80(8.20-16.30)	0.000
PLT, ×10^9^/L (M, IQR)	124.00(88.00-164.00)	133(71.00-187.00)	0.481
LYM, ×10^9^/L (M, IQR)	0.54(0.37-1.09)	0.64(0.33-1.17)	0.799
ALT, U/L (M, IQR)	32.20(16.70-56.60)	33.10(17.60-64.00)	0.672
AST, U/L (M, IQR)	36.00(20.00-67.90)	40.0(20.0-67.90)	0.400
Hemoglobing/L (M, IQR)	99.00(83.00-112.00)	96.00(75.00-113.00)	0.783
TBIL μmol/L (M, IQR)	10.20(6.90-16.50)	10.50(6.60-21.50)	0.914
LDH U/L (M, IQR)	267.4(185.10-371.80)	354.95(236.50-371.80)	0.021
BUN, mmol/L (M, IQR)	6.20(4.50-10.60)	8.38(5.65-14.00)	0.014
CR μmol/L (M, IQR)	78.80(55.90-108.50)	81.10(55.80-129.00)	0.547
PCT ng/ml (M, IQR)	0.72(0.36-4.39)	0.68(0.17-4.08)	0.290
D-dimer, mg/L (M, IQR)	2.39(1.52-3.54)	1.66(0.88-2.38)	0.016
ICU treatment			
MV (n, %)	39(90.69%)	61(85.92%)	0.563
Duration of MV days (M,IQR)	7(5.00-10.00)	7(4.00-10.0)	0.317
Length of ICU stay days (M,IQR)	10(7.00-19.00)	10.00(7.00-19.00)	0.421
28-day mortality (n, %)	14(32.56%)	19(26.76%)	0.529
SOFA (M, IQR)	11.00(9-12.00)	10(9.00-11.00)	0.213

WBC, White blood cell count; PLT, Platelet count; LYM, Lymphocyte count; ALT, Alanine aminotransferase; AST, Aspartate aminotransferase; TBIL, Total bilirubin; LDH, Lactate dehydrogenase; BUN, Blood urea nitrogen; CR, Creatinine; PCT, Procalcitonin; MV, Mechanical ventilation; ICU, Intensive care unit; SOFA, Sequential Organ Failure Assessment score; COPD, Chronic obstructive pulmonary disease; IQR, Interquartile range.

Compared with the non-neutropenic group, patients in the neutropenic group had significantly lower white blood cell counts (4.85 [3.50–7.45] × 10^9^/L vs. 12.80 [8.20–16.30] × 10^9^/L, P = 0.000) and a higher proportion of recent chemotherapy exposure (41.86% vs. 4.23%, P = 0.000). They also had higher body temperature (37.2 [36.8–38.0] °C vs. 37.0 [36.7–37.4] °C, P = 0.048), were younger (67 [58–77] vs. 76 [70–81] years, P = 0.001), and had a higher prevalence of hematologic diseases (51.16% vs. 29.57%, P = 0.028). In addition, D-dimer levels were significantly higher in the neutropenic group (2.39 [1.52–3.54] mg/L vs. 1.66 [0.88–2.38] mg/L, P = 0.016).In contrast, non-neutropenic patients exhibited a higher burden of chronic comorbidities, particularly chronic obstructive pulmonary disease (74.65% vs. 48.84%, P = 0.008) and Kidney disease (50.70% vs. 20.93%, P = 0.002). They also had higher levels of lactate dehydrogenase (354.95 [236.50–371.80] U/L vs. 267.40 [185.10–371.80] U/L, P = 0.021) and blood urea nitrogen (8.38 [5.65–14.00] mmol/L vs. 6.20 [4.50–10.60] mmol/L, P = 0.014) compared with the neutropenic group. Although SOFA scores were slightly higher in neutropenic patients, there were no statistically significant differences in SOFA scores or 28-day mortality between the two groups.

### 4.2 Comparison of the diagnostic performance of mNGS to CMTs in both groups

#### 4.2.1 Detection rate comparison between mNGS and CMTs

**Figure 2:**
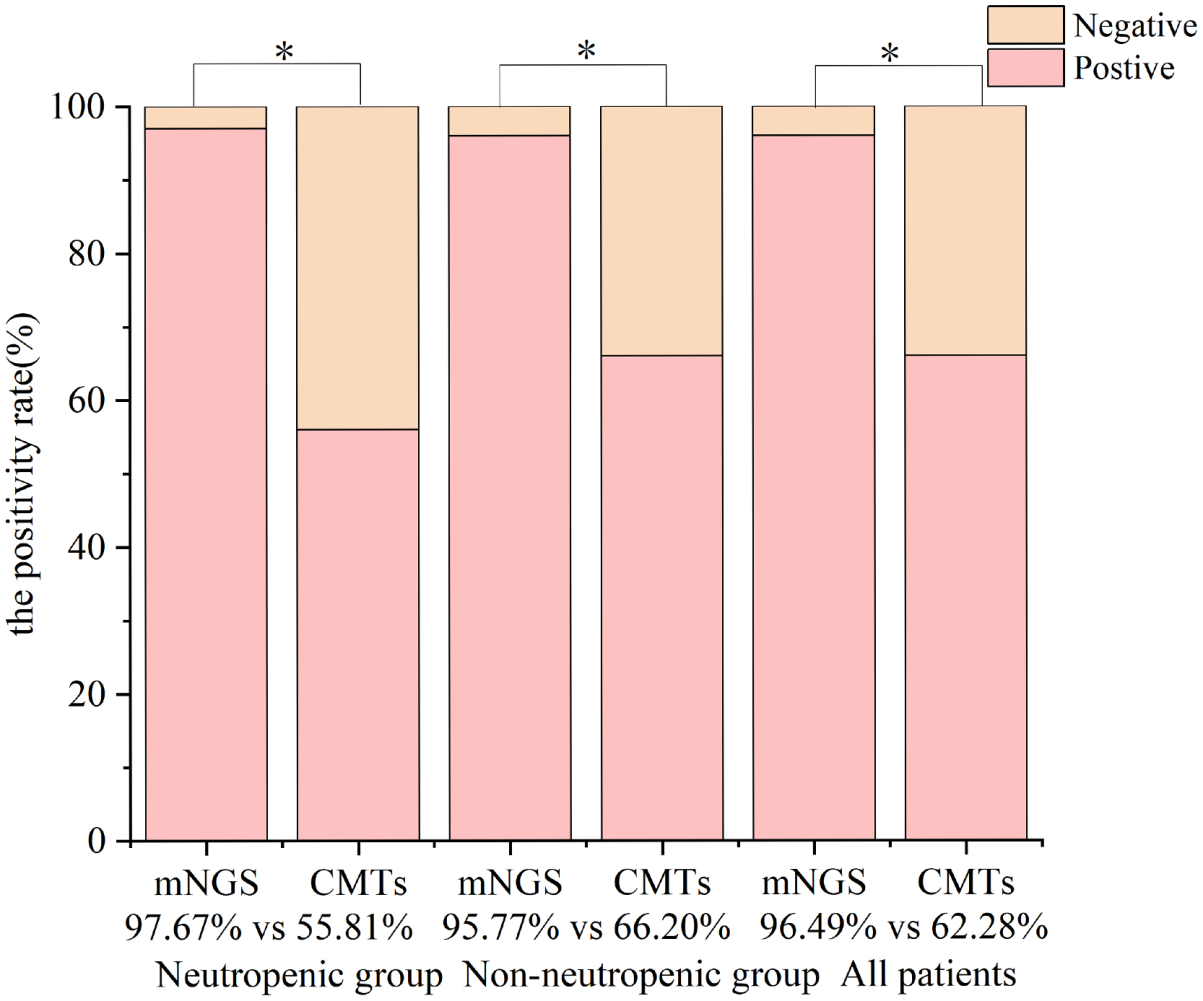
Comparison of positive detection rates between mNGS and CMTs in patients with IPA. Comparison of positive detection rates between metagenomic next-generation sequencing (mNGS) and conventional microbiological tests (CMTs) in patients with invasive pulmonary aspergillosis (IPA). *indicates P < 0.05.

All patients underwent both CMTs and mNGS of bronchoalveolar lavage fluid. Overall, the positivity rate of mNGS was significantly higher than that of CMTs (96.49% vs. 62.28%, P < 0.05). The higher mNGS detection rate was consistently observed in both neutropenic and non-neutropenic patients (**Figure 2**).

#### 4.2.2 Concordance between mNGS and CMTs

**Figure 3:**
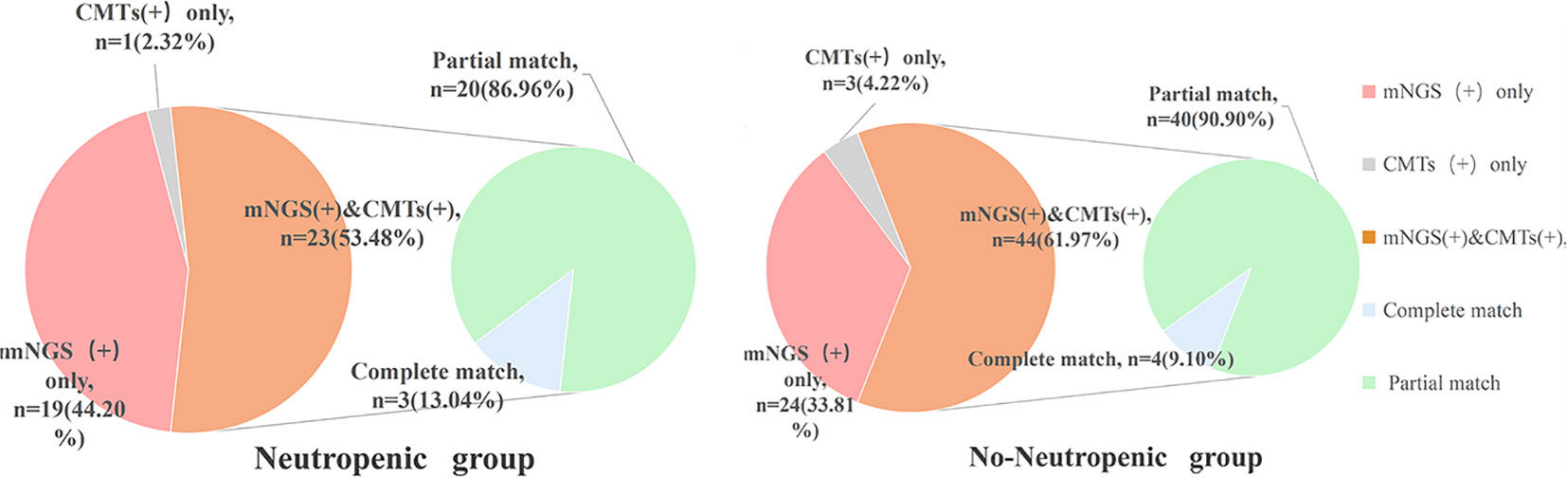
Distribution and concordance of pathogen detection results obtained by Mngs and CMTs in patients.

In the neutropenic group, both methods were positive in 53.48% of patients (23/43). mNGS alone was positive in 44.20% (19/43), and CMTs alone in 2.32% (1/43). Of the double-positive cases, complete concordance occurred in 13.04% (3/23), and partial concordance in 86.96% (20/23). In the non-neutropenic group, both methods were positive in 61.97% (44/71) of cases. mNGS alone detected pathogens in 33.81% (24/71), and CMTs alone in 4.22% (3/71). In double-positive cases, complete concordance was 9.10% (4/44), and partial concordance was 90.90% (40/44) (**Figure 3**).

#### 4.2.3 Pathogen spectrum and mixed infections by neutrophil status

mNGS demonstrated consistently superior pathogen detection across all categories compared with CMTs, regardless of neutrophil status. The most striking difference was observed in viral detection, where mNGS identified viruses in 53.48% (23/43) of neutropenic and 64.79% (46/71) of non-neutropenic patients, compared with only 2.33% (1/43) and 4.23% (3/71) by CMTs, respectively (both P < 0.05). Fungal detection rates were also markedly higher with mNGS in both the neutropenic (93.02% [40/43] vs. 55.81% [24/43]) and non-neutropenic groups (97.18% [69/71] vs. 63.38% [45/71]; both P < 0.05). A similar pattern was observed for bacterial detection (neutropenic: 65.12% [28/43] vs. 39.53% [17/43]; non-neutropenic: 66.20% [47/71] vs. 39.44% [28/71]; P < 0.05). Consequently, mNGS identified mixed infections at substantially higher rates than CMTs in both neutropenic (76.74% [33/43] vs. 27.91% [12/43]) and non-neutropenic patients (69.02% [49/71] vs. 22.54% [16/71]; both P < 0.05).

**Figure 4:**
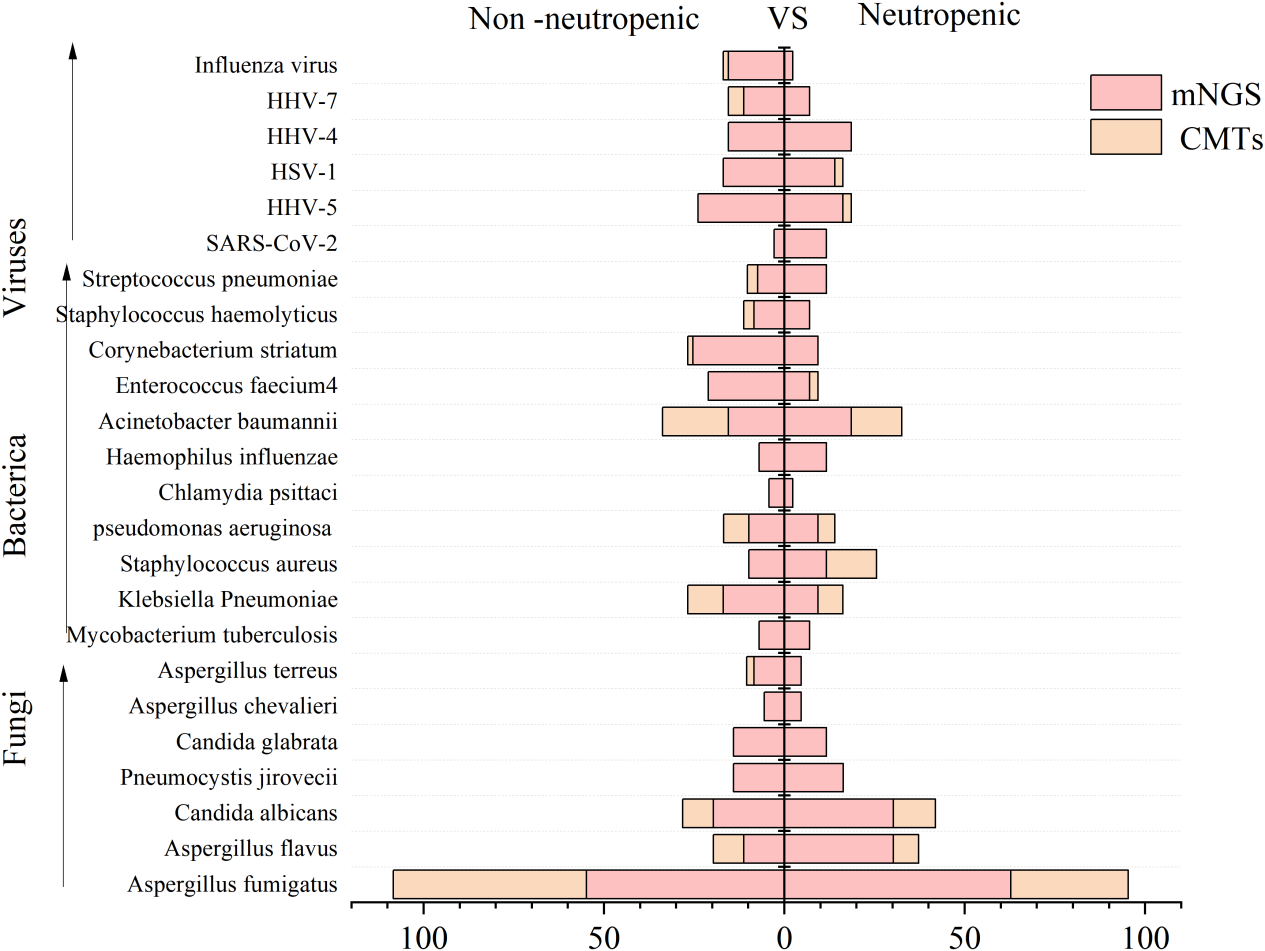
Overall distribution of pathogens in neutropenic and non-neutropenic patients.

As illustrated in **Figure 4**, the pathogen spectrum identified by mNGS differed markedly between neutropenic and non-neutropenic patients. Among bacterial pathogens, *Acinetobacter baumannii, Staphylococcus aureus*, and *Haemophilus influenzae* predominated in neutropenic patients, whereas *Corynebacterium striatum, Enterococcus faecium*, and *Klebsiella pneumoniae* were more frequently detected in non-neutropenic patients. Among fungal pathogens other than *Aspergillus spp, Candida spp.* and *Pneumocystis jirovecii* were the most identified organisms in both groups, with CMTs detecting only a limited range of additional fungal species. Regarding viral pathogens, Epstein–Barr virus (HHV-4), cytomegalovirus (HHV-5), and herpes simplex virus type 1 (HSV-1) were predominant across both groups; However, Human Herpesvirus 7 (HHV-7) and respiratory viruses, including influenza virus, were detected more frequently in non-neutropenic patients (**Figure 4**).

### 4.3 Relationship between mNGS-guided therapy and 28-day mortality

**Figure 5:**
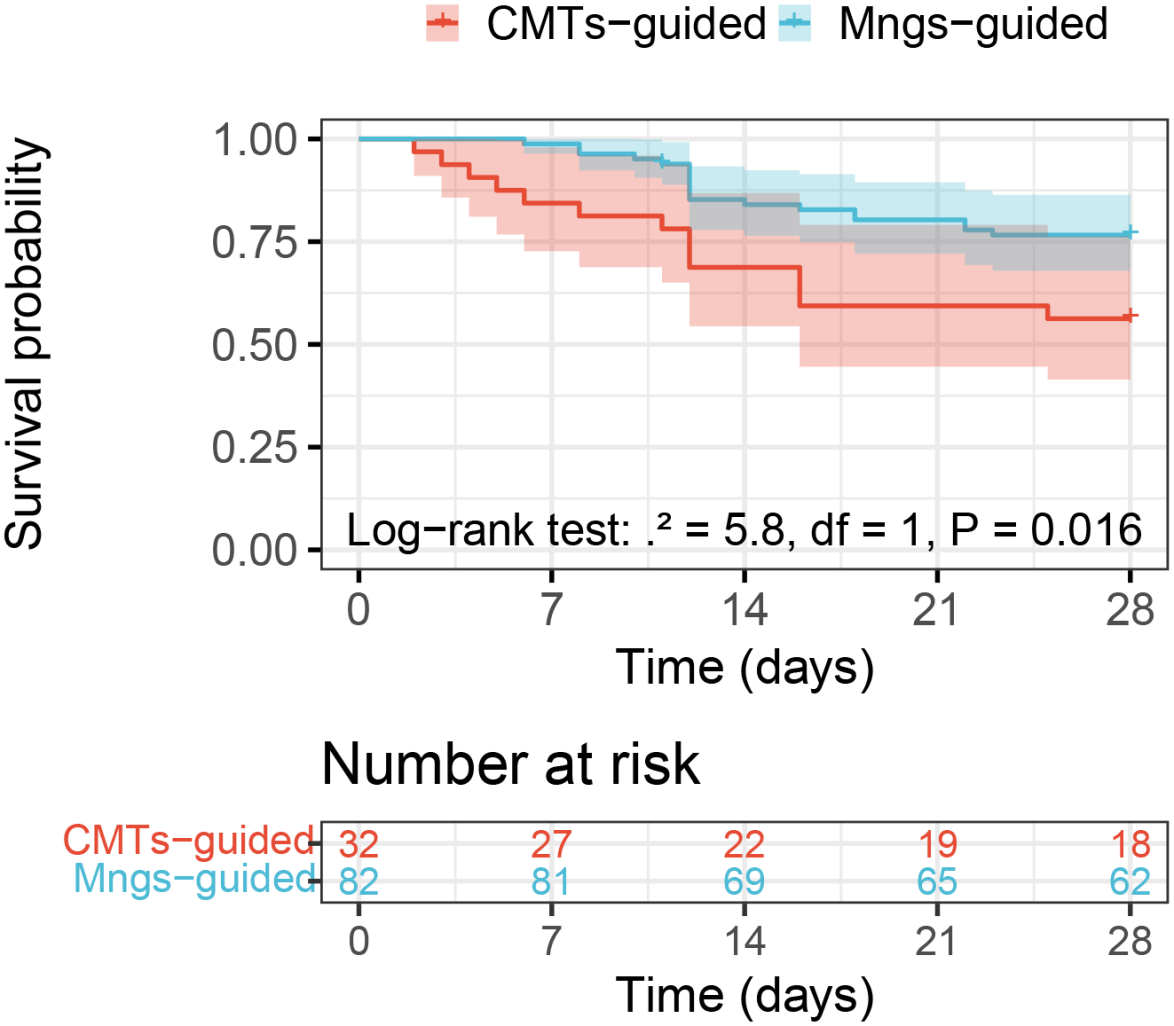
Kaplan–Meier survival curves comparing 28-day survival between mNGS-guided therapy and CMTs-guided therapy.

To further evaluate the clinical impact of mNGS-guided therapy, the baseline characteristics of patients in the mNGS-guided and CMTs-guided groups are presented in **Table 2**. Among the 114 patients who underwent antifungal therapy, 82 received mNGS-guided therapy, and 32 received CMTs-guided therapy. No significant differences were observed between the two groups in age, sex, SOFA score, mechanical ventilation, neutropenia status, Prior systemic antifungal therapy before BALF, comorbidity, and Clinical laboratory data (all P > 0.05) (**Table 2**). But mNGS-guided therapy was associated with a significantly lower 28-day mortality compared with CMT-guided therapy (23.17% [19/82] vs. 43.75% [14/32], P = 0.039). Consistently, Kaplan–Meier survival analysis demonstrated a significant survival benefit in the mNGS-guided group (log-rank test: χ² = 5.8, df = 1, P = 0.016; **Figure 5**).

**Table 2:** Baseline characteristics of patients with antifungal therapy guided by mNGS and CMTs.

Characteristics	mNGS-guided therapy (n=82)	CMTs-guided therapy(n=32)	P(value)
Age years (M, IQR)	72(69-78)	75(65-79)	0.485
Female (n, %)	12(14.63%)	8(25.00%)	0.272
Comorbidity			
Diabetes (n, %)	27(32.93%)	6(18.75%)	0.170
Hematologic disease (n, %)	31(37.80%)	12(37.50%)	0.976
Immunocompromised (n, %)	57(69.51%)	22(68.75%)	0.937
Neutropenia (n, %)	34(41.46%)	9(28.13%)	0.205
Clinical laboratory data			
WBC, ×10^9^/L (M, IQR)	9.90(5.15-14.85)	9.22(7.25-15.40)	0.564
PLT, ×10^9^/L (M, IQR)	133.00(75-185.50)	130.5(50-202.75)	0.925
LYM, ×10^9^/L (M, IQR)	0.63(0.38-1.15)	0.57(0.293-1.34)	0.821
Hemoglobin g/L (M, IQR)	95(78.25-110.75)	97.5(81.75-113.00)	0.652
TBL μmol/L (M, IQR)	11.70(8.20-19.75)	10.05(6.73-19.53)	0.485
BUN, mmol/L (M, IQR)	7.01(5.05-12.135)	7.51(5.57-12.07)	0.962
CR μmol/L (M, IQR)	77.90(55.80-116.60)	68.95(46.87-90.37)	0.113
D-dimer, mg/L (M, IQR)	1.94(1.15-3.16)	1.58(0.76-2.38)	0.161
PCT ng/mL (M, IQR)	0.95(0.16-5.00)	0.83(0.16-3.83)	0.632
ICU treatment			
MV, n(%)	75(91.46%)	25(78.13%)	0.063
Prior systemic antifungal therapy before BALF, (n, %)	15(18.29%)	5(15.63%)	0.736
Duration of MV days (M,IQR)	7(4.00-10.00)	7(4.00-10.00)	0.709
Length of ICU stay days (M,IQR)	9.50(6.00-17.00)	10.00(5.25-19.75)	0.645
28-day mortality (n, %)	19(23.17%)	14(43.75%)	0.039
SOFA (M, IQR)	11(9.00-12.00)	10(9-12)	0.689

**Figure 6:**
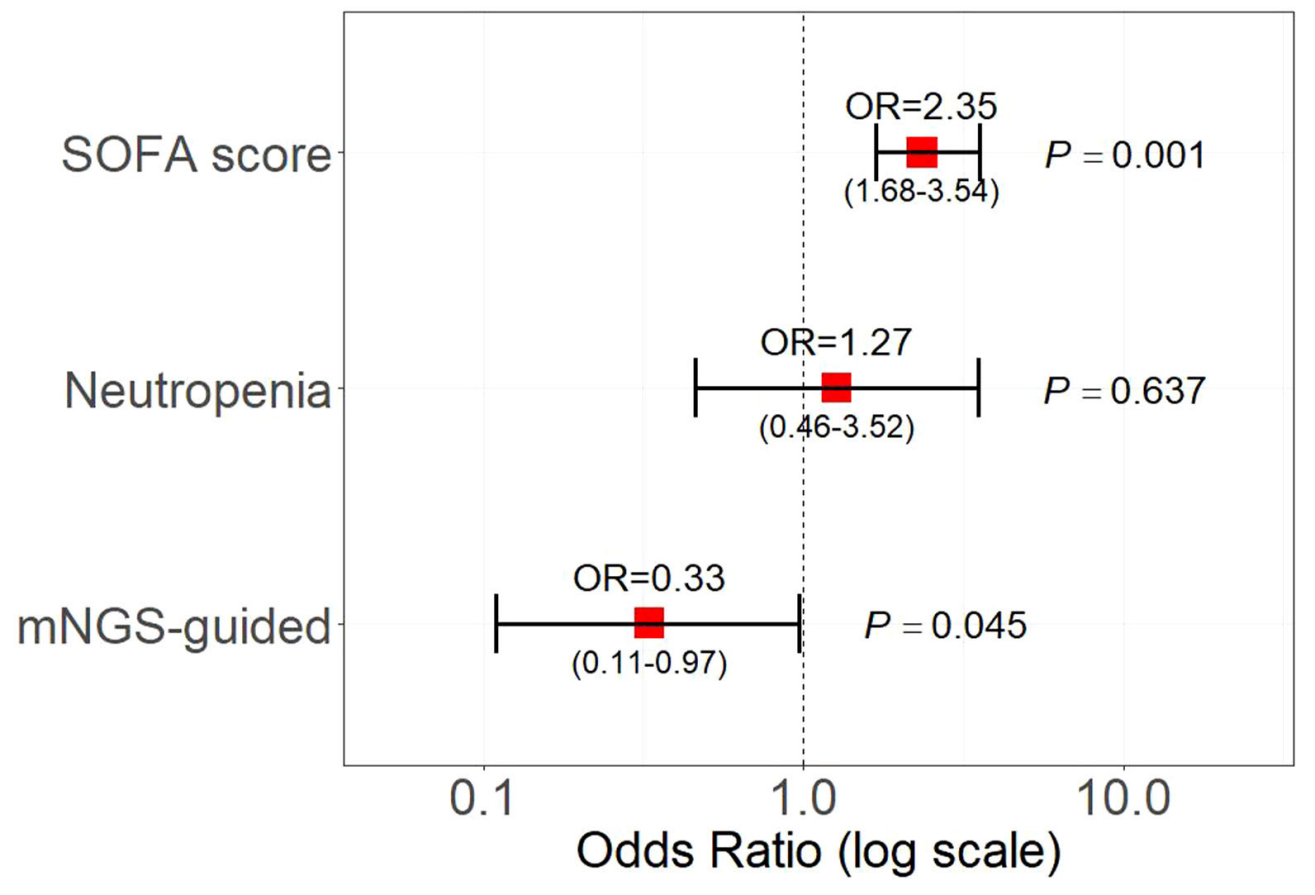
Multivariable logistic regression analysis of factors associated with 28-day mortality. Forest plot of multivariable logistic regression analysis for 28-day mortality. The model included SOFA score, neutropenic status, and mNGS-guided therapy. Data are presented as odds ratios (ORs) with 95% confidence intervals (CIs).

To identify factors associated with prognosis in patients with IPA, the baseline characteristics of survivors (n = 81) and non-survivors (n = 33) are presented in **Table 3**. No significant differences were observed between the two groups in age, sex, neutropenic status, immunocompromised status, or common comorbidities, including hypertension, diabetes, heart disease, chronic obstructive pulmonary disease, renal disease, and hematologic disease (all P > 0.05). However, non-survivors had significantly higher SOFA scores (P = 0.001) and a lower proportion of mNGS-guided therapy (P = 0.039) compared with survivors. Other laboratory parameters and comorbidities were comparable between the two groups (all P > 0.05) (**Table 3**). We performed univariate logistic regression to identify variables potentially associated with 28-day mortality. Variables with P < 0.1 in the univariate analysis were considered for inclusion in the multivariable analysis. Neutrophil status, although not meeting this threshold, was additionally included based on clinical relevance and it prespecified role in subsequent interaction analysis. To avoid over adjustment, a parsimonious multivariable logistic regression model was constructed, including mNGS-guided treatment, SOFA score, and neutrophil status, to assess the independent effect of mNGS-guided therapy on prognosis. Multivariable analysis showed that mNGS-guided therapy was independently associated with reduced 28-day mortality (adjusted OR = 0.329, 95% CI: 0.111–0.974, P = 0.045) (**Figure 6**).

**Table 3:** Comparison of baseline clinical characteristics between survivors and non-survivors.

Characteristics	Survivors (81)	Non-survivors(n=33)	P-value
Age years (M, IQR)	70.5(64.25-77.0)	73.0(69-80)	0.141
Female (n, %)	14(17.28%)	6(18.18%)	0.978
Fever (M, IQR)	37.00(36.80-37.85)	37.20(36.80-37.45)	0.987
Comorbidity			
Hypertension (n, %)	45(55.55%)	22(66.66%)	0.302
Diabetes (n, %)	23(28.39%)	10(30.30%)	0.824
Heart disease (n, %)	18(22.22%)	10(30.30%)	0.325
COPD (n, %)	51(62.96%)	23(69.70%)	0.525
Kidney disease (n, %)	29(35.80%)	16(48.48%)	0.291
After chemotherapy (n, %)	14(17.28%)	7(21.21%)	0.605
Hematologic disease (n, %)	27(33.33%)	16(48.48%)	0.142
Immunocompromised (n, %)	54(66.67%)	25(75.76%)	0.379
Disease severity			
SOFA (M, IQR)	8(7.00-10.00)	12(10.5-13.00)	0.001
Clinical laboratory data			
WBC, ×10^9^/L (M, IQR)	9.90(6.70-15.80)	9.40(5.83-13.58)	0.490
PLT, ×10^9^/L (M, IQR)	124.00(88.00-164.00)	133(71.00-187.00)	0.630
Neutropenia (n, %)	29(35.80%)	14(42.42%)	0.529
CRP μmol/L (M, IQR)	91.02(39.19-205)	109.32(41.74-192.95)	0.757
PCT ng/ml (M, IQR)	0.69(0.13-7.25)	0.36(0.10-2.34)	0.300
D-dimer, mg/L (M, IQR)	1.79(0.88-2.83)	2.77(1.10-2.83)	0.404
ICU treatment			
mNGS-guide (n, %)	63(77.8%)	19(57.58%)	0.039
MV (n, %)	70(86.42%)	30(90.90%)	0.754
Duration of MV (day)	7.0(4-10)	7.5(5.75-10.25)	0.164

#### 4.3.1 Impact of neutropenic status on clinical benefit of mNGS

**Figure 7:**
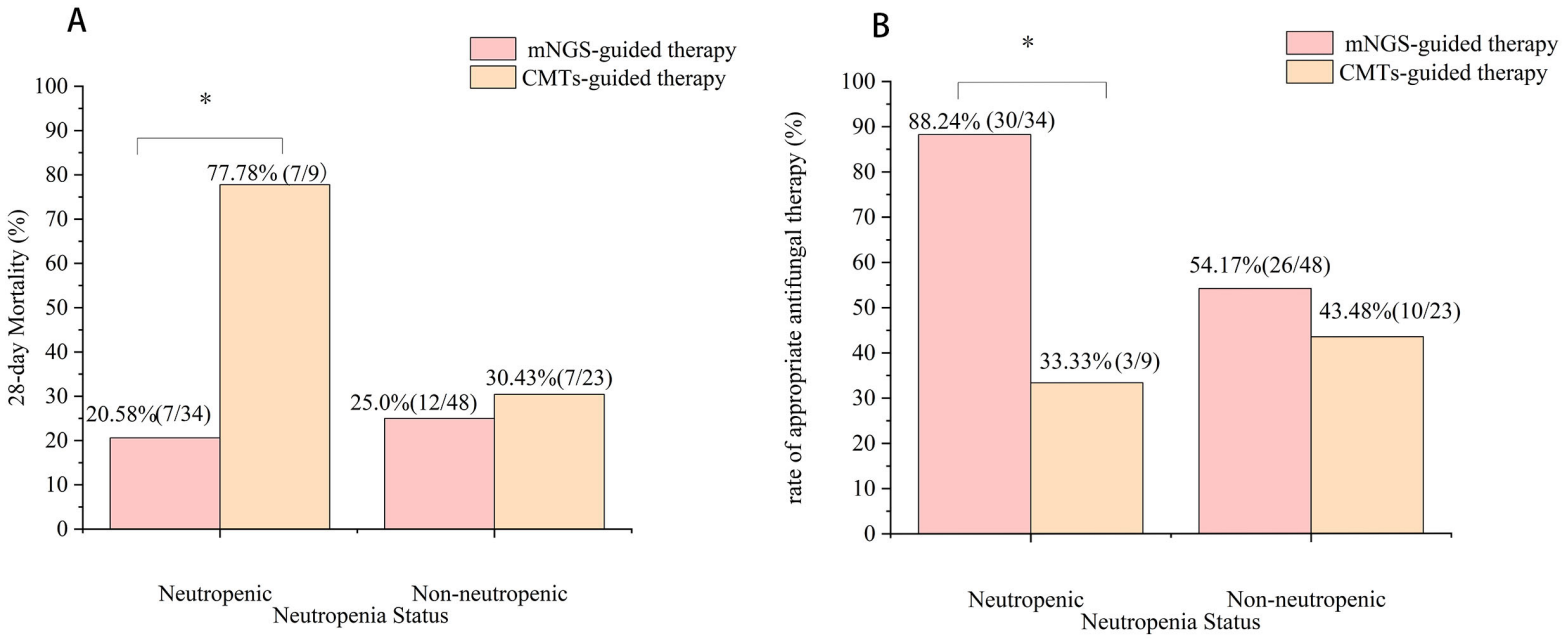
28-day mortality and Antifungal therapy in IPA subgroups. **(A)** Comparison of 28-day mortality between mNGS-guided therapy and CMTs-guided therapy in neutropenic and non-neutropenic patients. **(B)** Rate of appropriate antifungal therapy in neutropenic and non-neutropenic patients under mNGS-guided and CMTs-guided strategies. *Data are presented as raw counts/total subgroup patients, with corresponding percentages in parentheses (e.g., 77.8% (7/9)). Notably, the neutropenic CMTs-guided subgroup has a relatively small sample size. Statistical significance between groups is indicated as P < 0.05.

To assess effect modification by neutropenia status, an interaction term between neutropenia and mNGS-guided therapy was included in the multivariable model. After adjustment for confounding factors, the interaction was statistically significant (adjusted P = 0.008). Subgroup analysis revealed that the clinical benefit of mNGS-guided therapy was influenced by baseline neutropenic status (**Figure 7**). Among neutropenic patients, the 28-day mortality rate was significantly lower in the mNGS-guided group than in the CMTs-guided group (20.58% [7/34] vs. 77.78% [7/9]; P = 0.003). In contrast, no statistically significant difference in mortality was observed between the two diagnostic strategies in non-neutropenic patients (25.0% [12/48] vs. 30.43% [7/23], P = 0.775) (**Figure 7A**).

#### 4.3.2 Impact of mNGS on diagnostic efficiency and appropriateness of antifungal therapy

To further explore the potential mechanisms underlying the improved clinical outcomes associated with mNGS-guided therapy, we evaluated its impact from two complementary perspectives: diagnostic efficiency and the appropriateness of antifungal therapy. The mNGS group demonstrated a significantly shorter median TAT than the conventional testing group (32 [23–40] vs. 100 [26–115] hours, P < 0.01). The rate of appropriate antifungal therapy was significantly higher in the mNGS group than in the conventional testing group (65.17% [56/82] vs. 40.63% [13/32], P = 0.010). Further stratified analysis by neutropenic status revealed a significant effect modification: In the neutropenic subgroup, the mNGS group showed a significantly higher rate of appropriate antifungal therapy compared with the conventional testing group (88.24%[30/34] vs. 33.33%[3/9], P = 0.002); In the non-neutropenic subgroup, no statistically significant difference was observed between the two groups (54.17% [26/48] vs. 43.48%[10/23], P = 0.452) (**Figure 7B**).

#### 4.3.3 Sensitivity analysis using a temporal classification approach

A sensitivity analysis was performed using predefined temporal criteria. Two patients were reclassified, resulting in 80 patients in the mNGS-guided therapy group and 34 patients in the CMTs-guided therapy group. The association between mNGS-guided therapy and lower 28-day mortality remained consistent with the primary analysis (22.5% [18/80] vs. 44.1% [15/34], P = 0.025). Multivariable logistic regression analysis confirmed that mNGS-guided therapy was independently associated with reduced 28-day mortality (adjusted OR = 0.337, 95% CI: 0.116–0.975, P = 0.045).

## 5 Discussion

In this multicenter retrospective study, we found that mNGS-guided antifungal therapy significantly reduces 28-day mortality in patients with invasive pulmonary aspergillosis (IPA) compared with CMTs. Subgroup analysis further showed that this benefit was mainly observed in patients with neutropenia, while no significant difference was found in non-neutropenic patients. There was also a significant interaction between neutrophil status and the effect of mNGS-guided therapy.

Neutropenia is one of the most important host-related risk factors for IPA and reflects severe impairment of innate immune defense. In our cohort, non-neutropenic patients were older and had a higher burden of comorbidities, including COPD and chronic kidney disease, as well as higher white blood cell counts. Although the 28-day mortality was numerically higher in the neutropenic group, the difference was not statistically significant, consistent with previous studies (Bolger et al., 2014). Some studies have even reported similar or higher mortality in non-neutropenic patients (Cornillet et al., 2006). This paradox may be related to differences in clinical awareness and management strategies. Neutropenia often prompts earlier and more aggressive antifungal treatment, whereas immune dysfunction in non-neutropenic patients is more subtle, with non-specific clinical and imaging manifestations, potentially leading to delayed diagnosis or insufficient treatment intensity.

mNGS is an unbiased and culture-independent method capable of simultaneously detecting bacteria, fungi, viruses, and parasites. Consistent with previous studies (Zhao et al., 2025b; Zhao et al., 2025a; Zhao et al., 2025c; Zhao and Ye, 2025), mNGS showed a significantly higher overall positivity rate than CMTs in ICU patients with IPA, in both neutropenic and non-neutropenic subgroups. In addition, mNGS demonstrated superior performance in identifying mixed infections and atypical or fastidious pathogens, such as Mycoplasma, Pneumocystis jirovecii, and non-tuberculous mycobacteria.

Based on these diagnostic advantages, we further evaluated its clinical impact. mNGS significantly shortened pathogen detection time (median, 32 vs. 100 h; P < 0.01) and improved the appropriateness of antifungal therapy (65.17% vs. 40.63%, P = 0.01). Multivariable analysis confirmed that mNGS-guided therapy was an independent protective factor for 28-day mortality after adjustment for disease severity and neutrophil status. Although direct evidence in antifungal settings remains limited, our findings align with the broader concept that earlier molecular diagnosis may improve outcomes by accelerating pathogen identification and facilitating timely treatment adjustment (Sun et al., 2026). The observed benefit may therefore be partly attributed to reduced turnaround time and improved therapeutic precision.

However, the effectiveness of translating diagnostic information into clinical decision-making differed between host subgroups. In neutropenic patients, mNGS significantly increased the rate of appropriate antifungal therapy (88.24% vs. 33.33%, P = 0.002), suggesting a more direct and rapid impact on treatment decisions. In contrast, no such significant benefit was observed in non-neutropenic patients. Previous studies have shown that neutropenic IPA is more frequently associated with angioinvasive disease (Imaging Working Group for the Revision and Update of the Consensus Definitions of Fungal Disease from EORTC/MSGERC, 2021), characterized by rapid progression and a narrow therapeutic window, making early pathogen identification particularly critical.

Additionally, the positivity rate of CMTs in neutropenic patients was lower than in non-neutropenic patients (55.81% vs. 66.20%), consistent with previous hematological studies (Xu et al., 2023). This discrepancy may reflect low pathogen burden, prior exposure to broad-spectrum antibiotics, and the requirement of viable organisms for culture-based detection. These factors reduce the sensitivity of conventional methods and highlight the added value of mNGS in neutropenic populations (Jia et al., 2023; Chen et al., 2025).

Consistent with the above mechanistic rationale, neutropenic patients may derive greater benefit from mNGS-guided antifungal therapy, To further validate this hypothesis, multivariate regression analysis adjusted for potential confounders was performed. A significant interaction between neutropenia status and mNGS-guided antifungal therapy was identified, suggesting a potential survival benefit of mNGS guidance in neutropenic patients. However, interpretation of subgroup survival differences requires caution. Among 43 neutropenic IPA patients, only 9 received antifungal therapy primarily guided by conventional microbiological tests (CMTs). The observed mortality rate in this subgroup (77.78%) is subject to substantial statistical uncertainty due to the very small sample size and may be markedly influenced by the outcome of a single patient. Therefore, this estimate should not be overinterpreted. Importantly, the interaction effect obtained from the full multivariate model is credible, while the survival difference only seen among neutropenic patients is merely preliminary exploratory evidence. Larger prospective cohorts are needed to verify the real survival gap between the two treatment strategies in neutropenic populations.

Although mNGS also demonstrated clear diagnostic advantages in non-neutropenic patients, these did not translate into significant clinical benefits, as no differences were observed in 28-day mortality (25.0% vs. 30.4%, P = 0.775) or appropriate antifungal therapy rate (54.17% vs. 43.48%, P = 0.452). Two explanations may account for this finding. First, limited organ functional reserve may restrict the translation of diagnostic improvements into therapeutic gains. Non-neutropenic patients often have impaired hepatic and renal function (Morena et al., 2025; Terrones-Campos et al., 2026), as reflected in our cohort by a higher prevalence of chronic kidney disease (50.70% vs. 20.93%, P = 0.002) and elevated blood urea nitrogen levels (8.38 vs. 6.20 mmol/L, P = 0.014). According to antifungal guidelines, organ dysfunction often necessitates dose adjustment or treatment modification, which may limit the clinical utilization of mNGS-derived information.

Second, differences in disease phenotype may also play a role. Non-neutropenic patients more commonly present with airway-invasive rather than angioinvasive disease (Jenks et al., 2021), which tends to progress more slowly. In this context, the advantage of rapid pathogen identification may have a less immediate impact on short-term outcomes.

Furthermore, a high rate of mixed infections was observed in both groups (76.7% in neutropenic and 69.0% in non-neutropenic patients), highlighting the value of mNGS in detecting complex pathogen spectra (Liu et al., 2025). Immunocompromised patients are more prone to polymicrobial infections, while conventional methods are limited by narrow detection scope and low sensitivity (Zhao et al., 2024). mNGS can simultaneously detect bacteria, fungi, and viruses, making it particularly valuable in severe immunosuppression. In addition, distinguishing non-Aspergillus mold infections from Aspergillus is often difficult in clinical practice, yet their antifungal susceptibilities differ significantly (Park et al., 2011), further underscoring the clinical relevance of mNGS.

In summary, the diagnostic advantages of mNGS and its ability to optimize treatment decisions are associated with improved clinical outcomes in ICU patients with IPA, particularly in those with neutropenia. These findings suggest that the clinical benefit of mNGS is strongly influenced by host immunosuppressive status.

This study has several limitations. Although a multicenter retrospective design with multivariable adjustment was used, residual confounding and selection bias cannot be fully excluded. First, every patient took both mNGS and routine microbial tests, so we avoid bias from giving only one test to certain patients. However, different doctors may judge the two sets of test results differently, bringing slight residual confounding that fixed grouping rules cannot completely remove. Second, using mNGS as part of IPA diagnostic standards may cause mild selection bias in our cohort. Large prospective studies are still needed to confirm the clinical value of this combined testing plan (mNGS plus traditional microbial tests. In addition, IPA diagnosis was primarily based on clinical criteria rather than histopathological confirmation, as lung biopsy is rarely feasible in critically ill patients, which may introduce diagnostic heterogeneity. In addition, the neutropenic subgroup included patients with heterogeneous underlying diseases, and further stratified analyses were limited by sample size. Antifungal therapy in the CMTs group was guided by multiple diagnostic methods rather than a standardized protocol, potentially introducing treatment variability. Although mNGS has high sensitivity, colonization and contamination cannot be fully excluded, and cost-effectiveness was not assessed. Finally, only patients who received antifungal therapy were included; however, untreated patients accounted for only 2.63% of the cohort and are unlikely to have significantly influenced the overall conclusions.

## Data Availability

The data presented in the study are deposited in the GSA‑Human in the National Genomics Data Center (https://ngdc.cncb.ac.cn) repository, accession number HRA019992 (BioProject: PRJCA069599).
